# A Repurposing Strategy for Hsp90 Inhibitors Demonstrates Their Potency against Filarial Nematodes

**DOI:** 10.1371/journal.pntd.0002699

**Published:** 2014-02-13

**Authors:** Victoria Gillan, Kerry O'Neill, Kirsty Maitland, Francis M. Sverdrup, Eileen Devaney

**Affiliations:** 1 Institute of Biodiversity, Animal Health and Comparative Medicine, University of Glasgow, Glasgow, United Kingdom; 2 Center for World Health & Medicine, Saint Louis University, Saint Louis, Missouri, United States of America; Michigan State University, United States of America

## Abstract

Novel drugs are required for the elimination of infections caused by filarial worms, as most commonly used drugs largely target the microfilariae or first stage larvae of these infections. Previous studies, conducted *in vitro*, have shown that inhibition of Hsp90 kills adult *Brugia pahangi*. As numerous small molecule inhibitors of Hsp90 have been developed for use in cancer chemotherapy, we tested the activity of several novel Hsp90 inhibitors in a fluorescence polarization assay and against microfilariae and adult worms of *Brugia in vitro*. The results from all three assays correlated reasonably well and one particular compound, NVP-AUY922, was shown to be particularly active, inhibiting Mf output from female worms at concentrations as low as 5.0 nanomolar after 6 days exposure to drug. NVP-AUY922 was also active on adult worms after a short 24 h exposure to drug. Based on these *in vitro* data, NVP-AUY922 was tested *in vivo* in a mouse model and was shown to significantly reduce the recovery of both adult worms and microfilariae. These studies provide proof of principle that the repurposing of currently available Hsp90 inhibitors may have potential for the development of novel agents with macrofilaricidal properties.

## Author Summary

Adult filarial worms are long-lived nematode parasites that have proved very difficult to kill with existing drugs. Current campaigns for the control or elimination of these parasites are largely based on treatment with drugs such as diethylcarbamazine or ivermectin, that preferentially kill the first stage larvae of the parasite, the microfilariae. As microfilariae repopulate the body from unaffected adult worms, repeated dosing with these drugs is required over the long reproductive life span of the worm. The availability of compounds with macrofilaricidal activity would help facilitate the goal of controlling filarial infections. Hsp90 is a recognized target in tumor cells: consequently many oncology programs have developed small molecule inhibitors of Hsp90, several of which are commercially available. Here we provide proof of principle that inhibition of Hsp90 is lethal to adult *Brugia* worms *in vivo*, as well as *in vitro*, suggesting that these compounds may have potential for further development as macrofilaricidal drugs.

## Introduction

Infections caused by the parasitic filarial nematodes *Wuchereria bancrofti*, *Brugia malayi* and *Onchocerca volvulus* remain a significant cause of pathology in the tropics. The adult stages of these pathogens are extremely difficult to kill with currently available drugs. Treatment relies upon two compounds, ivermectin (IVM) or diethylcarbamazine (DEC), both of which largely target the larval stage of the life cycle (the microfilariae, Mf). In the Global Campaign for the Elimination of Lymphatic Filariasis, either DEC or IVM is combined with albendazole. While this approach effectively disrupts transmission [1], Mf repopulate the circulation, necessitating the repeated administration of drug. As the reproductive life span of the adult female worm is estimated to be around 10 years for the lymphatic species [2] and longer for *Onchocerca volvulus* [3], programs aimed at eradication of these parasites are faced with a considerable challenge, as treatment must be continued over this long timescale. At least for *O. volvul*us, the repeated administration of ivermectin over many years is associated with treatment failures [4], although whether these truly reflect resistance remains the subject of debate. Consequently, drugs that target adult filarial worms would be a major advantage in control programs aimed at eliminating these parasites [5].

Heat shock protein 90 (Hsp90) has emerged in recent years as a validated target for the therapy of various tumors [6], resulting in the development of many Hsp90-specific small molecule inhibitors. Hsp90 is essential in all eukaryotes and several recent studies have demonstrated the activity of specific inhibitors against a variety of tropical pathogens, such as *Plasmodium* [7], [8], *Trypanosoma* sp [9] *Leishmania* sp [10] and the filarial worm *Brugia* [11], [12]. The repurposing of compounds designed for one purpose to control of tropical infections is an attractive proposition [13], generating considerable enthusiasm in the pharmaceutical industry. Starting the search for new therapeutics for these diseases with drug-like molecules offers several short cuts, as these have already passed the basic criteria for development, have usually been optimized for their drug-like qualities and have often undergone toxicity testing.

Here we compare the efficacy of several classes of Hsp90 inhibitor against the lymphatic filarial nematode *Brugia*. The prototype Hsp90 inhibitor is geldanamycin (GA), a fermentation product of *Streptomyces* species that binds at the N-terminal ATP domain of Hsp90 disrupting its function [14]. Hsp90 acts as a molecular chaperone helping to fold and stabilize a variety of different proteins, the so-called ‘client’ proteins, many of which are involved in signal transduction [6]. The realization that Hsp90 client proteins, such as those encoded by oncogenes, were unable to attain their active conformation and were degraded following exposure to GA led to studies in animal models of various cancers. However, GA suffers from several target-unrelated limitations as an *in vivo* chemotherapeutic agent because of its chemical structure, as it contains a benzoquinone ring, rendering it hepatotoxic [15]. GA has been extensively modified to limit these liabilities and some of the resulting derivatives are still undergoing clinical assessment (reviewed in [16]). However, most recent efforts have been directed at developing synthetic small molecule inhibitors of distinct chemical scaffold, such as the purine-scaffold series [17], that bind at the same site as GA but lack the target-unrelated liabilities. These molecules have undergone considerable modification and one compound, PU-H71, shows potential in the clinic [18], [19]. Several additional N-terminal Hsp90 inhibitors have been identified in high throughput screens, including the pyrazole, isoxazole and triazole resorcinol classes such as VER-50589, NVP-AUY922 and STA-9090 (ganetespib), respectively [20], [21]. NVP-AUY922 is progressing through Phase I/II clinical trials while STA-9090 has advanced to Phase III [22], [23]. An additional class of compound, the Serenex series, also progressed to phase I/II clinical trials (reviewed in [22]). In this paper we report the efficacy of five inhibitors, representing four different classes of compound, on adult *Brugia in vitro* and compare the results with those from screens based on Mf viability and a fluorescence polarization assay. We focus on the most active compound, NVP-AUY922, and describe its *in vitro* effects on three life cycle stages of *Brugia* and its efficacy against adult worms *in vivo*.

## Methods

### Ethics statement

All animal protocols were carried out in accordance with the guidelines of the UK Home Office, under the Animal (Scientific Procedures) Act 1986, following approval by the University of Glasgow Ethical Review Panel. Experiments were performed under the authority of the UK Home Office, project numbers 60/4448 and 60/3792.

### Parasites

The *Brugia pahangi* life cycle was maintained by serial passage through mosquitoes (*Aedes aegypti*, Refm) and jirds, *Meriones unguiculatus*, as described previously [24].

### Preparation of worm extracts

Adult worms of *B. pahangi* were obtained from infected jirds after 3–4 months, exactly as described previously [11] and were frozen in liquid nitrogen, ground in a pestle and mortar to a fine powder and re-suspended in an appropriate volume of HFB assay buffer (20 mM HEPES, pH 7.3, 50 mM KCl, 5 mM MgCl_2_, 20 mM Na_2_MoO_4_, 1% NP40). Protein concentrations were estimated using the BioRad protein assay. At this point lysates were freeze-dried for shipping to the USA.

### Fluorescence polarization assay

The FP assay was set up essentially as described previously [12], [25]. In brief, assays were performed in black 96-well half-volume non-binding microtiter plates (Corning #3686) in a total volume of 50 µl per well. Assay buffer (HFB2) contained 20 mM HEPES, pH 7.3, 50 mM KCl, 2 mM EDTA, 0.01% Triton-X100, 0.1 mg/ml bovine gamma globulin (Sigma #G5009, Saint Louis, MO), 2 mM DTT (Sigma, Saint Louis, MO) and protease inhibitor cocktail (Roche #11836170, Indianapolis, IN). The equilibrium binding of Cy3B-GA and recombinant human Hsp90α (Enzo Life Sciences, Farmingdale, NY USA) or parasite lysate was determined by creating a two-fold dilution series of protein/extract for an eleven-point curve with the first column containing no protein. The dilution series was incubated with 6 nM Cy3B-GA in triplicate at 4°C with gentle shaking for different periods of time and FP measurements taken on a Safire2 plate reader (Tecan, San Jose, CA) with excitation and emission wavelengths of 530 nm and 585 nm, respectively, and a bandwidth of 20 nm. All FP values are expressed in millipolarization (mP) units with the mP value of free Cy3B-GA probe set to 50. Equilibrium binding constants were determined by nonlinear regression using a one-site binding model (GraphPad Prism software).

### Competition FP assays

The relative binding affinities of inhibitors to human or parasite-derived Hsp90 was determined using competitive FP binding assays. Human Hsp90 was used at a concentration resulting in 50% maximal binding of 6 nM Cy3B-GA (2.4 nM for human Hsp90α). For parasite-derived Hsp90, an amount of parasite lysate resulting in 50% of maximal Cy3B-GA binding was used. The drugs tested in the FP assay were GA and 17-AAG, CCTC018159, VER-49009, VER-50589, NBP-AUY922, NVP-BEP800, CAY 10607, BIIB021, PU-H71, SNX-2112, SNX-9203 and HSP990. Stock solutions of each compound were prepared in DMSO at a concentration of 10 mM and 3-fold serial dilutions prepared in DMSO for eleven point curves. Drugs were then diluted 100-fold into HFB2 assay buffer containing 12 nM Cy3B-GA in 96-well storage plates to create 2X drug solutions. Drug solutions (25 µl/well) were then transferred in duplicate to 96-well black assay plates (Corning#3686) containing 25 µl HFB2 with 2X the final desired concentration of Hsp90. The final concentration of Cy3B-GA was 6 nM and the final DMSO concentration in all wells was 0.5%. Free Cy3B-GA (mP set to 50) and buffer only (background) wells were included as controls on each plate. Plates were incubated at 8°C with gentle shaking for 20 h. FP measurements were taken and the inhibitor concentration at which 50% of bound Cy3b-GA was displaced (IC_50_) was determined using nonlinear regression with a four parameter logistic equation (GraphPad Prism software).

### Drugs

The five new compounds selected for *in vitro* testing on *B. pahangi* were NVP-AUY922, NVP-BEP800, SNX-2112, SNX-9203 and BIIB021. GA was used as a positive control in some experiments. All compounds were supplied by Selleck Chemicals (www.selleckchem.com), with the exception of GA, which was supplied by MBL International Corporation (Woburn, MA). Drugs were dissolved in DMSO to give a stock solution of 10 mM, then aliquoted and stored at −20°C. Working concentrations of drugs were prepared on the day of use by dilution in tissue culture medium. For *in vivo* studies, NVP-AUY922 was purchased from LC Laboratories (www.LCLabs.com) and dissolved in DMSO at 50 mg/ml.

### Effect of inhibitors on *B. pahangi* viability *in vitro*

As adult *Brugia* worms are limited in numbers, initial experiments assessed the effect of each drug on Mf viability. Mf were purified from infected animals essentially as described previously [26]. In brief, following lavage of the peritoneal cavity of an infected animal with Hanks Balanced Salt Solution (HBSS) pre-warmed to 37°C, Mf were collected by centrifugation and then purified from host cells by centrifugation through lymphoprep (Sigma). This procedure was repeated twice. Mf were collected from the pellet, washed twice in HBSS and once in worm culture medium (WCM) which comprised RPMI 1640, (Invitrogen Cat No: 52400), containing 5% heat inactivated fetal calf serum, 1% glucose, 100 units/ml penicillin and 100 µg/ml streptomycin (all Invitrogen). Mf were then dispensed into the wells of a 24-well plate to give approximately 200 Mf in 2.0 ml, using a single well for each drug concentration. All procedures were carried out using aseptic techniques. The five novel compounds, plus GA and medium alone controls, were tested three times against Mf over the full range of concentrations.

For adult worm assays, adult female *B. pahangi*, 3–4 months old, were incubated individually in 2.0 ml of WCM overnight in 24-well plates and pre-screened for Mf production. Any worms that failed to produce Mf overnight were discarded. For the drug experiments, six female worms of *B. pahangi* for each concentration of drug were cultured individually in 24-well plates in 2.0 ml of WCM containing drug, or carrier alone (DMSO) at a concentration equal to that in the highest concentration of drug. In some experiments, GA was used as a positive control. Initially, all five compounds were tested over selected concentrations starting at 2 µM to 100 nM (see Results for details). For Hsp90 inhibitors, Mf output by individual female worms is a sensitive indicator of adult worm viability and, in most experiments, was assessed at 72 h. In addition, adult worms were examined microscopically on a daily basis for 7–10 days to determine whether lower concentrations of drugs had any effects over a longer period of incubation. Results are expressed as mean Mf output ± SD over a 72 h period. Statistical significance between groups was calculated using the Mann Whitney test with P values<0.05 being considered significant.

In two additional experiments, adult worms were exposed to a short 24 h incubation in 250, 25 or 10 nM NVP-AUY922 or DMSO in medium alone, using six worms per concentration as described above. After 24 h in drug, adult worms were removed, washed out of drug and incubated in medium alone. Mf output was counted after 24 h in drug and again after 24 h or 48 h in medium alone. Plates were maintained for up to 10 days and the condition of adult parasites noted at regular intervals. In one experiment the effect of GA on Mf output by adult *B. malayi* worms was compared with *B. pahangi*. *B. malayi* worms were kindly provided by Prof. R. Maizels (University of Edinburgh). In this experiment, Mf were counted after 48 h of culture in GA at 2.0 µM and 1.0 µM GA. In all *in vitro* experiments, plates were viewed daily and the motility and condition of the parasites noted by two independent observers, of whom one was unaware of the contents of the well.

L3 stages were harvested from mosquitoes nine days post-infection. L3 were picked individually with a fine glass pipette, counted and washed three times in HBSS containing 1000 units/ml of penicillin and 1000 µg/ml streptomycin by sedimentation at room temperature. 20–30 L3 per well were plated out in duplicate in 24-well plates in 2.0 ml of WCM containing drug, or carrier alone (DMSO) and cultured at 37°C for up to 7 days. NVP-AUY922 was tested at a range of concentrations: 500, 250, 100, 50, 25, 10, 5.0, 1.0, 0.5 and 0.1 nM. This experiment was repeated twice.

### *In vivo* testing of NVP-AUY922

Adult worms were removed from the peritoneal cavity of infected jirds and rinsed in HBSS. 10 adult female worms were transplanted into the peritoneal cavity of each of ten male BALB/c mouse using standard methods [27], [28]. Adult worms were transferred into the peritoneal cavity of anaesthetized mice using a blunted glass hook. Not all mice received the full quota of 10 worms (see Table 1). The stock solution of drug was diluted to 5 mg/ml in sterile PBS containing Tween 20, to a final concentration 5%, and DMSO to a final concentration of 10%, as detailed previously [29]. Five mice were treated with 50 mg/Kg NVP-AUY922 and five with sterile PBS/Tween 20/DMSO by intra-peritoneal injection at three time points: day 0 (7 days post-transplantation), day 3 and day 7. This dose was selected on the basis of previous studies in a mouse xenograft model [29]. Adult worms and Mf were recovered 9 days after the last dose of drug by peritoneal lavage with pre-warmed HBSS. The condition of any adult worms recovered was noted. Mf in the first 12 ml of peritoneal washings were pelleted by centrifugation and fixed in 2% formalin in water and stored at 4°C until counted. Mice were weighed at each time point to monitor any possible weight loss.

**Table 1:** Recovery of adult worms and Mf from mice post-NVP-AUY922 treatment.

Group	Treated	Control
No. mice with worms	5/5	5/5
No. mice with viable worms	2/5	5/5
Total number worms recovered	8/48**	26/47**
No. viable worms recovered (mean % ± SD)	2 (4.4±6.0)	24 (51.6±18.5)
No. mice with live Mf	2/5	5/5
Mean no Mf per mouse	4730±5101	45,880±17687

** not every animal received the full complement of 10 female worms.

Recoveries from individual drug-treated mice: 3/10; 1/10; 2/9; 1/9; 1/10.

Only 2 of the 8 worms recovered were normal, all others were coated in cells and largely immobile

Recoveries from individual control animals: 8/9; 3/10; 4/10; 6/10; 5/10. All worms recovered from control animals showed normal motility and had no adherent cells with the exception of a single worm, in each of two mice.

## Results

### A fluorescence polarization (FP) assay shows binding of various classes of Hsp90 inhibitor to both *B. malayi* and *B. pahangi* and to human Hsp90α

Several classes of inhibitor were screened against *B. pahangi or B. malayi* lysates, as well as human Hsp90α, using the FP assay originally developed as a high throughput screen for Hsp90 inhibitors in tumor cells, as previously applied to *Brugia* [12]. This assay is based on the ability of small molecules to inhibit the binding of Cy3B labeled GA to Hsp90. Table 2 shows the range of IC_50_ values for the binding of selected compounds to *B. pahangi*, *B. malayi* or human Hsp90α. In general, most compounds bound to parasite-derived and human Hsp90 with broadly similar affinities. Several compounds bound worm Hsp90 with high affinity, including NVP-AUY922 and VER-50589, each of which bound *Brugia* Hsp90 at low nanomolar concentrations (IC_50_ 1–2 nM). A second group of compounds including GA, PU-H71, SNX-2112, SNX-9203, BIIB021 and VER-49009 bound *Brugia* Hsp90 with an IC_50_ in the range of 10–20 nM. Most drugs bound to Hsp90 from both species of *Brugia* with broadly similar affinity; CAY10607 was an exception in this respect, showing a 10-fold higher affinity for *B. pahangi* Hsp90 compared to *B. malayi* Hsp90.

**Table 2:** Binding affinity of various Hsp90 inhibitors versus human Hsp90α, *B. pahangi* and *B. malay*i Hsp90 in the FP assay.

Compound	HsHsp90α IC_50_ (nM)^a^	*B. pahangi* IC_50_ (nM)^a^	*B. malayi* IC_50_ (nM)^a^
Geldanamycin	8.7 (7.1–11)	6.4 (5.3–7.8)	12 (9.5–15)
17-AAG	12 (8.6–18)	8.0 (7.1–9.0)	2.5 (2.0–3.3)
CCT018159	270 (190–400)	240 (180–320)	330 (280–390)
VER-49009	8.0 (5.8–11)	11 (8.1–15)	14 (11–17)
VER-50589	1.5 (1.3–1.8)	0.54 (0.40–0.72)	1.2 (0.99–1.4)
NVP-AUY922	1.1 (1.0–1.4))	0.64 (0.42–1.0)	0.95 (0.68–1.3)
CAY10607	2.2 (1.7–2.8)	1.6 (1.3–2.0)	16 (12–22)
NVP-BEP800	13 (8.7–18)	6.9 (3.7–13)	8.7 (6.2–12)
BIIB021	2.6 (1.7–4.2)	16 (14–18)	14 (12–17)
PU-H71	10 (6.6–15)	17 (14–21)	27 (22–34)
SNX-2112	4.6 (3.5–6.0)	15 (6.7–35)	20 (13–33)
SNX-9203	3.8 (2.8–5.0)	10 (5.3–19)	11 (7.1–18)
HSP990	1.4 (1.1–1.8)	nd	2.3 (1.6–3.3)

a Best fit (95% confidence intervals).

nd = not determined.

### Pre-screening using Mf identifies a spectrum of drug activity

**Figure 1:**
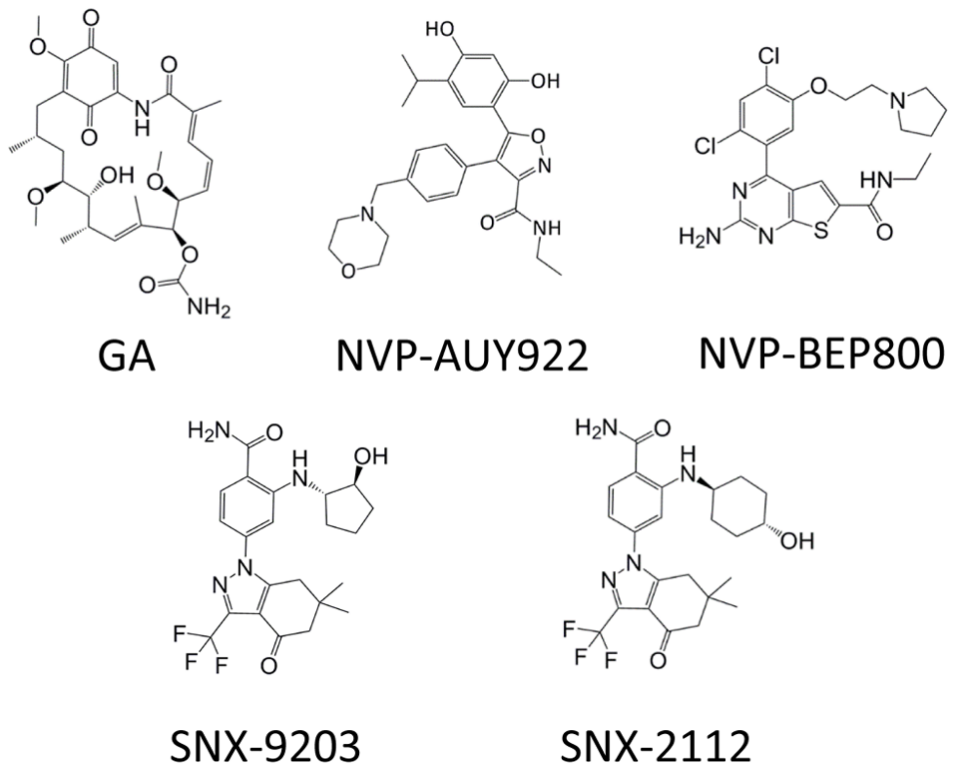
Structures of Hsp90 inhibitors used in the *in vitro* experiments. Figure shows chemical structures of Geldanamycin (GA), NVP-AUY0922, NVP-BEP800, SNX-9203 and SNX-2112.

We initially compared the efficacy of Hsp90 inhibitors against Mf as this life cycle stage is extremely abundant, while the numbers of adult worms are more restricted. In these experiments six drugs, representing five different chemotypes were selected including NVP-AUY922, NVP-BEP800, SNX-2112, SNX-9203, BIIB021 and GA (see Fig. 1 for drug structures), were tested over ten doubling dilutions from 4.0 µM to 0.976 nM and compared with the DMSO vehicle. The results of these experiments were clear-cut: NVP-AUY922 was by far the most effective compound tested, killing 100% of Mf by day 7 at all concentrations down to and including 1.95 nM. At the lowest concentration of NVP-AUY922 tested (0.976 nM), ∼75% of the Mf were dead after 7 days incubation. In contrast, a concentration of 500 nM NVP-BEP800 was required to kill the majority of Mf by day 7, with ∼50% death at 250 nM. Lower concentrations of NVP-BEP800 affected Mf motility but did not kill substantial numbers of worms. For the SNX compounds, SNX-2112 killed approximately 50% of Mf at 500 nM by day 7, while at 250 nM, Mf were very sluggish but still alive. SNX-9203 was slightly more effective at lower concentrations, killing ∼90% of Mf at 500 nM after 7 days exposure, while at 250 nM most worms were alive but were much less motile than controls. Finally, BIIB021 was active only at 4.0 µM, while lower concentrations affected worm motility but did not kill the worms within a 7-day period. In comparison, GA killed ∼90% of the Mf at 31.25 nM, while at 15.6 nM ∼50% of the Mf died, similar to the effective dose reported previously [11]. Thus, NVP-AUY922 was the most efficient inhibitor of Hsp90 as judged by Mf killing followed by GA, while BIIB021 was the least effective and the SNX compounds and NVP-BEP800 had broadly similar effects on Mf viability (see Table 3 for summary).

**Table 3:** Minimal effective doses of various compounds on Mf viability.

Drug	Concentration	% death by day 7
NVP-AUY922	1.95 nM	100%
	0.976 nM	75%
NVP-BEP800	250 nM	50%
SNX-9203	500 nM	90%
SNX-2112	500 nM	50%
BIIB021	4.0 µM	100%
GA	31.25 nM	90%
	15.6 nM	50%

Mf were cultured in 24-well plates in 2.0 ml medium containing drug at doubling dilutions from 4.0 µM to 0.976 nM. Plates were scored daily by microscopy and the percentage of worms alive estimated at each time point. Results are representative of three separate experiments.

### NVP-AUY922 affects Mf output by adult worms at low nanomolar concentrations

We have previously described a sensitive assay to record the effect of GA and the purine scaffold inhibitors on adult worm viability that involves counting Mf output over a designated time of exposure to inhibitor [11], [12]. In initial experiments, all five new compounds were screened against adult female worms at concentrations ranging from 2.0 µM to 100 nM and Mf output and adult worm viability monitored over a 7-day period (data not shown). Three of the five compounds tested (NVP-AUY922, NVP-BEP800 and SNX-9203) had a significant effect on Mf output at all doses tested including 100 nM, while SNX-2112 was significant only at 500 nM and BIIB021 at 2.0 µM. In additional experiments, NVP-BEP800 and the SNX compounds were further titrated from 500 nM to 1.0 nM to estimate the minimal effective concentration. For all three drugs a concentration of 500 nM was required to consistently show a significant effect on Mf output by adult worms in replicate experiments.

**Figure 2:**
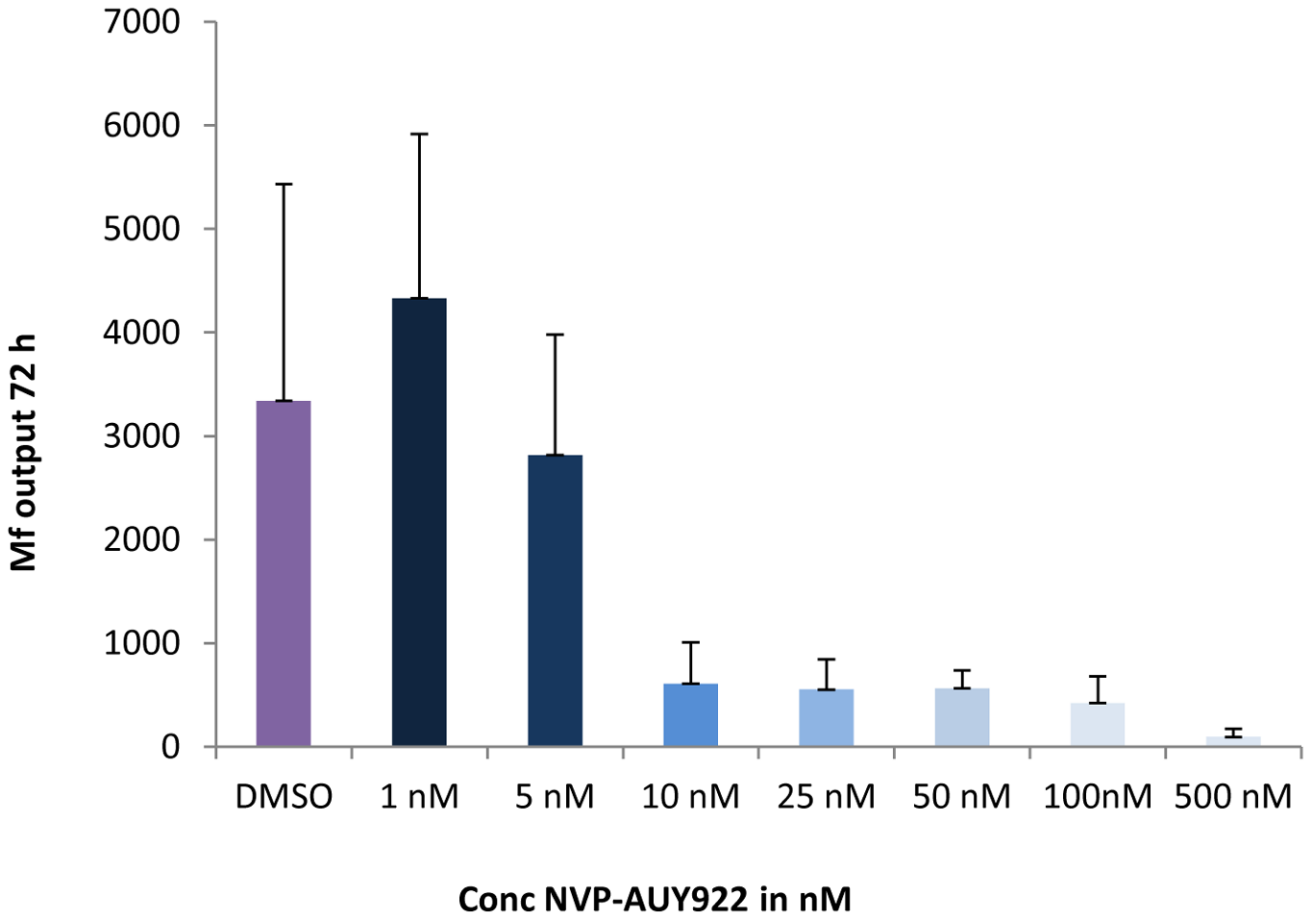
NVP-AUY922 significantly reduces Mf output from female worms at 10 nM. Graph shows mean (± SD) Mf output from groups of six female *B. pahangi* worms after 72 h exposure to NVP-AUY922 at 500, 100, 50, 25 10, 5 and 1 nM compared to DMSO control. Mf output is significantly different from control at all concentrations to 10 nM NVP-AUY922 (P≤0.0087). Results shown are representative of three experiments. In this experiment, cultures were continued for a further 3 days and Mf counted again. At this time point (a total of 6 days exposure to drug), Mf output was significantly different from controls at 5 nM NVP-AUY922 (P = 0.0043) but not at 1 nM.

Further experiments dealt only with the most effective compound NVP-AUY922, which was titrated over a range of concentrations from 500 nM to 1.0 nM. Following 72 h of exposure to all concentrations from 500 nM to 10 nM NVP-AUY922, a significant inhibitory effect on Mf output was observed (P = 0.0087 for 10 nM vs DMSO, see Fig. 2). Continuing the cultures for a further three days in the presence of drug resulted in a significant decrease in Mf output at 5.0 nM (P = 0.0043) but not at 1.0 nM. By 6 days of exposure all adult worms were dead at concentrations of 100 nM NVP-AUY922 and above, and, in a typical experiment (shown in Fig. 2), 4/6 worms were dead at 50 nM drug and 3/6 were dead at 25 nM drug. The remaining worms although alive, were extremely sluggish. Although the motility of the adult worms was affected by exposure to lower concentrations of NVP-AUY922 (10 and 5.0 nM), they did not die at these concentrations. Worms that were dying tended to burst and release their uterine contents and wells contained many embryonic stages in addition to Mf. Confirmation that NVP-AUY922 has a direct macrofilaricidal effect was obtained by exposing male worms to a range of concentrations from 5.0 µM to 100 nM. By day 7 of exposure 100% of male worms exposed to 100 nM drug were dead.

### NVP-AUY922 affects the L3 stage of *B. pahangi*

While for human filarial parasites, drugs that target adult worms are the priority, it was of interest to determine whether NVP-AUY922 also killed the infective form of the parasite, the L3. In these experiments, L3 were harvested directly from mosquitoes, washed and exposed to varying concentrations of NVP-AUY922 from 500 nM to 0.1 nM in WCM. After 6 days exposure to drug, 100% of L3 were dead at all concentration down to and including 10 nM. At 5.0 nM approximately 30% of parasites were dead and the remainder were moving very slowly. At this time point there was no significant mortality in control wells and, at concentrations of 1 nM or below, no effect on the L3 was observed. Thus NVP-AUY922 is toxic to L3 stages at relatively low concentrations.

### NVP-AUY922 is effective against adult worms following a short-term exposure *in vitro*

**Figure 3:**
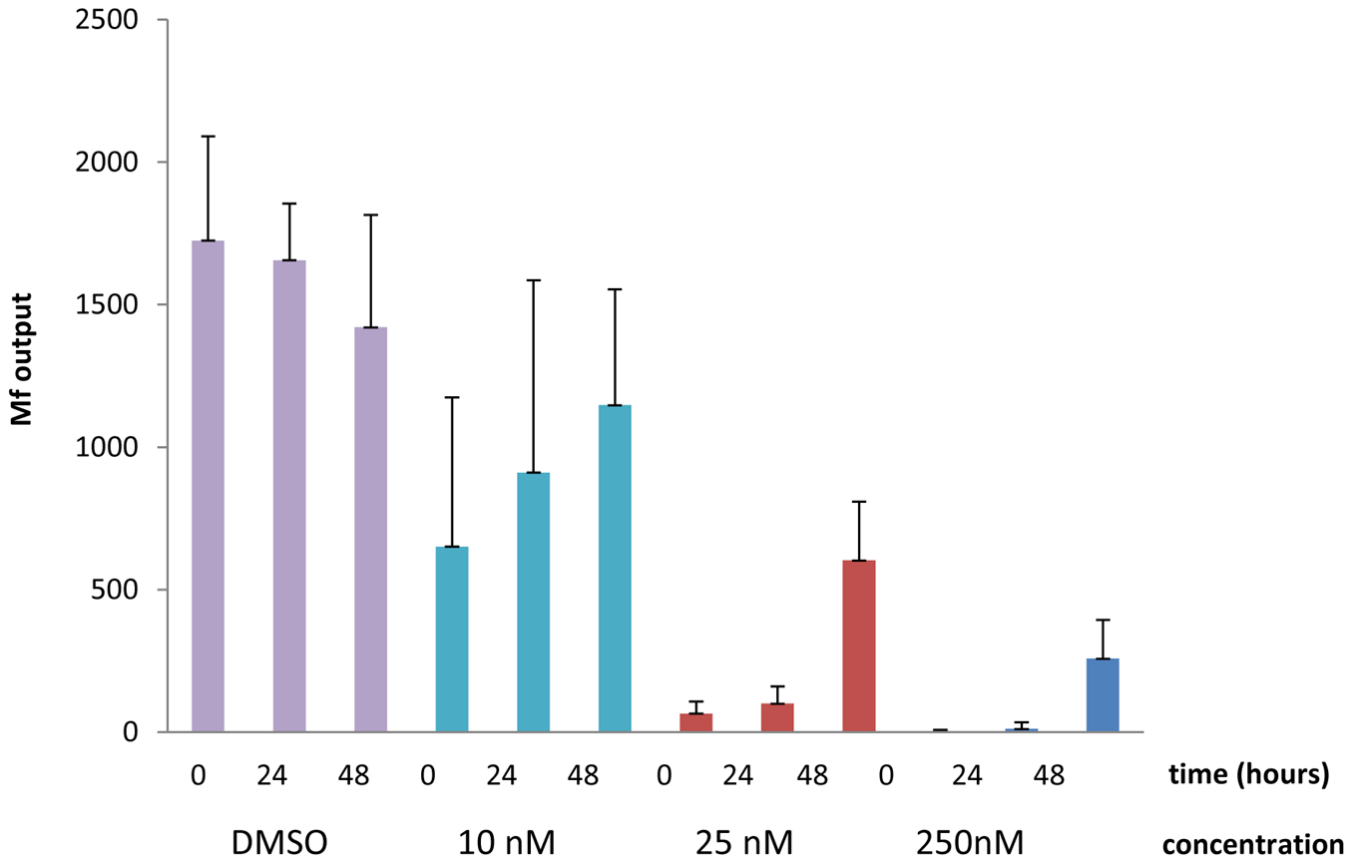
A 24-AUY922 reduces Mf output from female worms. Graph shows the mean (± SD) Mf output from groups of six female *B. pahangi* worms exposed to 250 nM, 25 nM or 10 nM NVP-AUY922 for a period of 24 h only (time 0). Worms were washed free of drug and cultures continued in medium alone for a further 24 h or 48 h and Mf output quantified. P<0.05 for 250 nM and 25 nM compared to control at all time points; for 10 nM drug, P<0.05 for 48 h only. Results shown are representative of two experiments.

In the experiments described above, adult worms or Mf were continuously exposed to drug and viability assessed. However, as any drug that might be used against filarial worms *in vivo* would be required to exert its effect over a limited period of time, we carried out two additional experiments to determine the outcome of exposing adult female worms to NVP-AUY922 for a 24 h period only. In these experiments six adult female worms per group were cultured individually with 250, 25 or 10 nM NVP-AUY922 or the appropriate concentration of DMSO alone for 24 h. Worms were then washed free of drug and cultured in medium alone for a further 7–9 days. After 24 h in drug, significantly fewer Mf were produced at 250 nM and at 25 nM NVP-AUY922 (P = 0.0043 for both concentrations) but no significant difference was observed in Mf output in worms incubated with 10 nM drug. After 24 h in drug and 24 h in medium alone, Mf production was almost completely inhibited from worms cultured in 250 nM drug (mean of 11±23 Mf) or 25 nM drug (mean of 100±60 Mf) compared to DMSO controls (1658±198, P<0.05 for both 250 nm and 25 nM drug). Although worms exposed to 10 nM drug for 24 h produced fewer Mf (mean 910±674 Mf) than DMSO controls this difference failed to reach significance (P = 0.0635). However following 24 h in drug and 48 h in medium alone, adult worms exposed to 10 nM NVP-AUY922 for 24 h produced significantly fewer Mf than control worms (P = 0.0317) (see Fig. 3). Adult worms were clearly affected by a short-term exposure to 250 nM NVP-AUY922, being much less motile than control worms after 24 h in medium alone. By 48 h they were elongate and by 9 days in medium alone, they were barely moving, but they were still alive. Worms exposed to 25 nM NVP-AUY922 were also noticeably more sluggish than control worms at day 9, while no obvious difference was observed between those incubated in 10 nM drug and DMSO controls. The experiment was discontinued at this point.

### *B. malayi* and *B. pahangi* adult females are equally sensitive to inhibition of Hsp90

Most of our experiments have been carried out using *B. pahangi*, a close relative of the human parasite, *B. malayi*. We compared the efficacy of GA at two concentrations (1.0 and 2.0 µM) on *B. malayi* in parallel experiments with *B. pahangi*. Not surprisingly, given the degree of amino acid identity between Hsp90 in the two species (93.5% identical), both were equally sensitive to Hsp90 inhibition. In this experiment, the reduction in Mf output at any one concentration of drug was almost identical: at 2.0 µM GA, there was a 93% reduction in Mf output in *B. malayi* and 92% with *B. pahangi* while at 1.0 µM, there was a 74% reduction in Mf output with *B. malayi* and a 78% reduction with *B. pahangi* after 48 h of drug exposure (P = 0.0043 for all concentrations of GA versus DMSO control).

### Testing NVP-AUY922 *in vivo*

As NVP-AUY922 appeared to be extremely active at low concentrations after a short exposure *in vitro*, it was pertinent to determine whether it would have activity *in vivo* against adult worms transplanted into the peritoneal cavity of BALB/c mice. Three animals in the treated group were given 10 adult worms, while the remaining two mice received 9 worms, while in the control group, one animal received 9 worms, one received 8 worms and the remaining three mice received 10 worms. Following adult worm transplant, mice were randomly assigned to a treatment group (five per group) and received either 50 mg/Kg NVP-AUY922 at three time points by intra-peritoneal injection or an injection of PBS/Tween 20/DMSO. Mice were weighed at each treatment and prior to recovery of adult worms, but no weight loss was observed in drug-treated animals over the time course of the experiment. Adult worms were recovered 9 days after the last injection of drug, at which point there was a significant reduction in worm recovery from treated mice compared to control animals (P = 0.0109) (see Table 1). All control animals contained live, motile adult worms with recoveries varying from 30–78% of transplanted worms (see Table 1 for details). In contrast, very few live worms were recovered from NVP-AUY922 treated mice (recovery of live worms ranged from 0–11%, Table 1). Adult parasites recovered from all animals were placed in HBSS at 37°C for 2–3 hours and examined again. There was no evidence to suggest that the adult worms recovered from drug-treated animals regained their motility over this time period. In three out of five treated animals, only a few dead Mf were observed in the peritoneal washings, while the remaining two animals contained low numbers of Mf that were very slow moving (P = 0.0079, NVP-AUY922 versus control).

## Discussion

In this paper we extend our observations on the effect of Hsp90 inhibitors on adult *Brugia* worms *in vitro* and *in vivo*. A panel of commercially available Hsp90 inhibitors, all designed and optimized for binding to human Hsp90, was profiled in FP binding assays. The broadly similar binding affinities between human and parasite proteins highlight the evolutionarily conserved structure of the nucleotide-binding domain that is targeted by these inhibitors. Selectivity towards parasite Hsp90 would obviously be preferable, but this endeavor would require a structure-based design effort, as recently described for trypanosome Hsp83 (Hsp90) [30]. In the spirit of exploring the potential for a direct repurposing strategy, the translation from parasite Hsp90 binding to filaricidal activity was examined. Five clinically viable compounds belonging to four different drug classes were tested *in vitro* for their ability to kill Mf and inhibit Mf output from adult female worms. The results from all three systems were reasonably consistent and highlight the efficacy of one specific inhibitor, NVP-AUY922. This compound showed significant activity against adult female worms at a concentration of 25 nM after 6 days exposure, significantly inhibiting Mf output and killing 50% of adult worms. Although lower concentrations of NVP-AUY922 (down to 5.0 nM) showed a significant effect on Mf output, adult worms were not killed at this concentration over the time scale of the experiment. NVP-AUY922 showed a high affinity for *Brugia* Hsp90 in the FP screen, further validating the application of this assay as a high throughput screen. It was also the most active compound tested in the Mf killing assay. As a single infected animal can produce millions of Mf, these results indicate that using Mf could provide a simple, inexpensive and relatively high throughput pre-screen for compounds with macrofilaricidal activity, while acknowledging that not all compounds that effect Mf will also kill adult worms. The translation from *Brugia* Hsp90 binding to micro- and macro-filaricidal activity depends upon the chemotype being tested and this is evident in the compounds used here. While NVP-AUY922 bound *Brugia* Hsp90 at ∼1 nM and had an EC_50_ against Mf viability in the same range, the other compounds exhibited variable translation between the two assays. For example, NVP-BEP800 bound quite well to *Brugia* Hsp90 (∼7 nM IC_50_) but required much higher concentrations to kill microfilaria (∼250 nM EC_50_) during an extended 7-day incubation. There are many possible explanations for this discrepancy, including failure of the compound to fully equilibrate within the microfilariae during the experiment. It is possible that this particular compound does not penetrate well or is actively exported from the parasites. As more detailed studies would be required to address this issue, we continued to focus on compounds with the best activity against live parasites.

We assessed the effect of these inhibitors on adult female worms by quantifying Mf output, as a measure of worm viability. Mf output is an active process in filarial worms and relies on the presence of live Mf *in utero* and on the activity of the vulva, a muscular opening close to the anterior end of the worm. When Hsp90 is inhibited, cessation of Mf output is a sensitive surrogate measure of adult female worm health and appears to be one of the first signs that worm viability is compromised. Once Mf production ceases following exposure to Hsp90 inhibitors, it does not resume, at least over the time scale of our *in vitro* experiments. In addition, previous studies [11] demonstrated that embryogenesis in adult *B. pahangi* was disrupted upon inhibition of Hsp90 with GA. When RNAi was used to knockdown *hsp90* (*daf-21*) in the free-living nematode *Caenorhabditis elegans*, one of the most penetrant phenotypes observed was a protruding vulva and sterility in the F1 generation [31]. In *C. elegans*, the vulva is a complex structure, the development of which is dependent upon a number of signaling pathways (reviewed in [32]). The vulva is innervated by neurons and egg-laying in *C. elegans* (the equivalent of Mf release in filarial worms) is regulated by multiple factors and molecules, including neuropeptides, kinases and members of the TGF-β signaling pathway [33].

Much less is known of the factors that regulate Mf production in filarial nematodes, but the vulva may be particularly sensitive to Hsp90 inhibitors, perhaps acting on various kinases required for signaling in this structure. Alternatively, the rapid inhibition of Mf output observed (within 24 h at high concentrations of inhibitor) may reflect a general demise in adult female worms upon inhibition of Hsp90. In the related filarial nematode *O. volvulus*, ivermectin is reported to inhibit Mf output, while not killing the adult worm. In cattle infected with *O. ochengi*, embryogenesis is disrupted by treatment with ivermectin and an accumulation of dead and dying Mf are observed [34], similar to that described for female *Brugia* exposed to GA [11]. However, the results of the present study show that inhibition of Hsp90 by NVP-AUY922 not only affects Mf release but also directly affects the viability of adult worms. This property was clearly demonstrated following *in vitro* exposure of both male and female adults to the inhibitor and *in vivo* administration of the inhibitor to mice following transplant of female worms. Hsp90 functions in a complex with other proteins to fold and/or stabilize a wide variety of client proteins, most of which have been identified from mammalian cells or yeast. A list of Hsp90 client proteins is curated by the Picard Lab (http://www.picard.ch). However, we know little about the key client proteins clients in *Brugia*, except by homology with known interacting proteins identified from other systems. A better understanding of the Hsp90 interactome in *Brugia* may help explain the effects observed upon chemical inhibition of Hsp90.

As all our previous studies have focused on *B. pahangi*, we also tested *B. malayi* in this study and showed that GA had very similar effects upon both species. Similar data was recently published [35] showing that GA and four derivatives of GA, were active *in vitro* on adult *B. malayi* and the trematode parasite *Schistosoma japonicum*. In that study, the lowest concentration tested was 500 nM, but encouragingly all compounds were active against adult *B. malayi* at this concentration.

Interestingly, NVP-AUY922 also showed potent activity against the L3 stage of *B. pahangi*. While drugs that target the L3 stage of filarial nematodes are not the highest priority for human use, they are of significant interest in the veterinary field, where prophylaxis of the dog heartworm, *Dirofilaria immitis*, is a major area of concern in veterinary practice. Here, the macrocyclic lactones, such as ivermectin and moxidectin, have been the mainstay of control for many years. Given the recent observation on treatment failures with ivermectin in some dogs in the Southern states of the USA [36], there is a clear need for the development of novel compounds to protect susceptible animals.

As a preliminary to *in vivo* testing, we investigated whether a short exposure to the most effective compound identified, NVP-AUY922, affected adult worm viability *in vitro*. These experiments showed that Mf output was significantly reduced following a 24 h exposure to all concentrations of drug including 10 nM (the lowest concentration tested in this study), although it required an additional 48 h incubation in medium alone to detect this effect. However, although Mf output was significantly different between drug exposed and control worms, a direct effect was observed at 250 nM concentration after a 24 h exposure, with adult female worms being almost motionless after 9 days in culture. While a 24 h exposure to 25 nM NVP-AUY922 resulted in a significant decrease in motility, the adult female worms were still alive at day 9. Whether such motility-impaired worms would be viable *in vivo* in an immuno-competent host is doubtful. These data also highlight one of the characteristics of Hsp90 inhibitors: the time taken to kill adult worms. They do not kill rapidly, which may be a useful attribute for *in vivo* use, as some of the pathogenesis of lymphatic filariasis is associated with death of the adult worms, and the subsequent release of antigen and the Wolbachia endosymbiont [37].

NVP-AUY922 belongs to the isoxazole resorcinol class of Hsp90 inhibitors and has shown some promise as an anti-tumor agent in a mouse xenograft tumor model [29], [38]. In the first of these studies, pharmacokinetic analysis showed that the plasma levels of drug reached a maximum of 52,506 nM at 0.25 h post-dosing following a single dose of 50 mg/Kg delivered by the intra-peritoneal route. As a 24 h exposure to 250 nM NVP-AUY922 had a significant deleterious effect on adult female worms, we were encouraged to test this compound *in vivo*, using an adoptive transplant system in which viable adult worms are transplanted into the peritoneal cavity of mice [27], [28]. In previous experiments with NVP-AUY922 administered in a similar dosing regimen to nude mice containing xeno-grafted tumors, weight loss was observed [38]. However our immune-competent animals showed no loss in weight or other obvious deleterious effects over the time scale of the experiment.

Adult worms were recovered nine days after the last dose of drug, as the *in vitro* data indicated that it might take some time for the drug to act. Additionally, previous studies with suramin, one of the few compounds with activity against adult filarial worms, demonstrated that it took approximately 6 weeks to reduce adult worm recovery in the jird model [39]. A total of eight out of 48 transplanted female worms were recovered from drug treated animals, but of these only two were healthy with the others being coated in cells, indicating that they were in the process of being cleared by the immune system. In contrast, 26 female worms out xof 47 transplanted were recovered from animal treated with vehicle alone. Moreover, only two of the drug-treated mice contained live Mf, and these appeared very sluggish compared to controls. Further studies will be required to examine alternative routes of drug administration and to explore in more detail the mechanism by which these inhibitors exert their macrofilaricidal effect.

The clinically viable Hsp90 inhibitors tested here are targeted and potent towards human Hsp90 and thus may have unwanted side effects. In this respect, the therapeutic potential of any of these inhibitors for filarial disease will depend on the pharmacodynamic differences between host toxicities and parasite killing. There are pharmacokinetic data in both animals and humans available for many of the Hsp90 inhibitors. We have used this data in combination with *in vitro* worm killing assays to predict the doses of NVP-AUY922 that would be successful in killing parasites *in vivo*. While efficacy in cancer treatment likely requires chronic dosing of near maximum tolerated doses, the treatment of parasitic diseases would ideally necessitate a short course of treatment. It is not currently clear whether a short treatment course with current inhibitors would be able to clear parasites, while maintaining appropriate safety margins. Additional studies will be required to carefully delineate the therapeutic window of short treatment courses. As noted earlier, the development of parasite-selective inhibitors with reduced potential for host toxicity would be of enormous benefit and should also be pursued.

The ubiquitous nature of Hsp90 in normal as well as transformed cells, has led some to question the potential of this molecule as a drug target. However, for the past 20 years, the National Cancer Institute has advocated Hsp90 as a drug target, since GA was first shown to exhibit anti-tumor properties. There are currently seventeen Hsp90 inhibitors in clinical trials and a growing arsenal of novel Hsp90 inhibitors, of structurally diverse scaffold (reviewed in [40]). Current emphasis is on combination therapies in which Hsp90 inhibitors are combined with other anti-tumor drugs [41]. Whether a similar approach would further enhance the activity of these inhibitors against adult filarial parasites remains to be determined. However, we believe that these studies provide data to further strengthen the contention that inhibition of Hsp90 is a valid target for the chemotherapy of lymphatic filariasis, while acknowledging that a significant medicinal chemistry effort would be required to optimize the activity of these inhibitors.
